# Asymptotic iteration method for solving Hahn difference equations

**DOI:** 10.1186/s13662-021-03511-9

**Published:** 2021-07-30

**Authors:** Lucas MacQuarrie, Nasser Saad, Md. Shafiqul Islam

**Affiliations:** School of Mathematical and Computational Sciences, University of Prince Edward Island, Charlottetown, Canada

**Keywords:** 39A05, 39A06, 39A13, 39A60, 47B39, Hahn operator, Linear difference equations, *q*-difference equations, Polynomial solutions, Eigenvalue problems

## Abstract

Hahn’s difference operator $D_{q;w}f(x) =({f(qx+w)-f(x)})/({(q-1)x+w})$, $q\in (0,1)$, $w>0$, $x\neq w/(1-q)$ is used to unify the recently established difference and *q*-asymptotic iteration methods (DAIM, *q*AIM). The technique is applied to solve the second-order linear Hahn difference equations. The necessary and sufficient conditions for polynomial solutions are derived and examined for the $(q;w)$-hypergeometric equation.

## Introduction

Hahn [1, 2] introduced, for $x\neq w/(1-q)$, the quantum difference operator

1 $$\begin{aligned}& D_{q;w}f(x) = \frac{f(q\, x+w)-f(x)}{(q-1)\,x+w},\quad q\in (0,1), w>0, \\& D_{q;w}^{0} f(x) =f(x),\qquad D_{q;w}^{n} f(x)= D_{q;w} \bigl\{ D_{q;w}^{n-1}f(x) \bigr\} ,\quad n=1,2,\ldots , \end{aligned}$$

where *f* is a function defined on an interval *I* of $\mathbb{R}$ which contains $w/(1-q)$. It is clear, using L’Hopital’s rule, that

$$ \lim_{x\to {w}/({1-q})}D_{q;w}f(x) =f^{\prime } \biggl( \frac{w}{1-q} \biggr) $$

provided that the function *f* is differentiable at $w/(1-q)$ in the usual sense.

The Hahn difference operator $D_{q;w}$ unifies (in the limit) and generalizes two well-known difference operators; namely, the quantum *q*-difference operator $D_{q}\equiv D_{q;0}$ (see [3–7]) and the forward difference operator $\Delta _{w}\equiv D_{1;w}$ (see [8, 9]). Included as a special case is the discrete forward difference operator $\Delta =D_{1;1}$. In the limit, both $D_{q}$ and $\Delta _{w}$ generalize the derivative operator $f'(x)=df/dx$. The generalization and the limit process to these important operators is illustrated by the following diagram:


A rigorous analysis of the calculus associated with the operator $D_{q;w}$ along with the construction of a proper inverse of $D_{q;w}$ and the associated integral calculus can be found in [10–12].

The Hahn operator $D_{q;w}$ is an important tool in the construction of families of orthogonal polynomials and several approximation problems (see, for example, [13–19]).

In 2003, Çiftçi, Hall, and Saad introduced [20, 21] the asymptotic iteration method (AIM) to solve analytically and/or approximately the second-order linear homogeneous differential equation

2 $$ y^{\prime \prime }(x) = \lambda _{0}(x)\, y^{\prime }(x) + s_{0}(x)\, y(x),\quad {}'=d/dx, $$

where $\lambda _{0}(x)$ and $s_{0}(x)$ are continuously differentiable functions. They proved that, up to some multiplication constant, an analytic solution of this differential equation reads

3 $$\begin{aligned} y(x) =\exp \biggl(- \int ^{x}\frac{s_{n-1}(t)}{\lambda _{n-1}(t)}\,dt \biggr) \end{aligned}$$

provided that, for some $n> 0$, (so-called terminating condition)

4 $$ \frac{s_{n}(x)}{\lambda _{n}(x)}= \frac{s_{n-1}(x)}{\lambda _{n-1}(x)}\quad \mbox{or} \quad \delta _{n}(x)= \lambda _{n}(x)s_{n-1}(x)-s_{n}(x) \lambda _{n-1}(x)\equiv 0, $$

given the AIM-sequences, for $n=0,1,2,\ldots $ , as

5 $$\begin{aligned}& \lambda _{n}(x) = \lambda _{n-1}^{\prime }(x) + \lambda _{n-1}(x) \lambda _{0}(x) +s_{n-1}(x), \\& s_{n}(x) = s^{\prime }_{n-1}(x) + s_{0}(x)\lambda _{n-1}(x), \end{aligned}$$

initiated with $\lambda _{-1}=1$ and $s_{-1}=0$. This powerful and simple technique [22] proved to be very useful in studying the eigenvalue problems in quantum mechanics. Noting that if the analytic solutions of the linear differential equation (2) are not available, the terminating condition (4) plays a vital role in approximating the exact solutions with high (and almost controllable) precision [23, 24]. Ismail and Saad [25] recently shed further insight on the AIM mathematical foundation where the reasons for its success and the possible convergence failures of the iterative aspect of it were presented.

In another study by Ismail and Saad [26], a discrete version of the AIM was presented, $dAIM$, along with applications to the classical difference equations [27]. Given the second-order linear difference equation

6 $$ \Delta ^{2}\, y(x)=\lambda _{0}(x)\, \Delta \, y(x)+s_{0}(x)\, y(x),\quad x\geq x_{0}, $$

a solution, up to some constant (periodic) function, is given by

7 $$ y(x)=\prod_{i=x_{0}}^{x-1} \biggl(1- \frac{s_{n-1}(i)}{\lambda _{n-1}(i) } \biggr) $$

provided that, for some $n>0$,

8 $$ \frac{s_{n}(x)}{\lambda _{n}(x)} = \frac{s_{n-1}(x)}{\lambda _{n-1}(x)},\quad n=1,2, \ldots , $$

where

9 $$\begin{aligned}& \lambda _{n}(x) =\Delta \, \lambda _{n-1}(x)+\lambda _{n-1}(x+1) \lambda _{0}(x)+s_{n-1}(x+1), \\& s_{n}(x) =\Delta \, s_{n-1}(x)+ \lambda _{n-1}(x+1)s_{0}(x). \end{aligned}$$

Also, the quantum *q*-discrete version $qAIM$ was introduced in [26]. Given a second-order *q*-difference equation

10 $$ D_{q}^{2}\, y(x)=\lambda _{0}(x)\,D_{q} y(x)+s_{0}(x)\, y(x),\quad 0< q< 1, $$

a solution, up to some constant periodic function, is given by

11 $$ y(x) ={\prod_{k=0}^{\infty } \biggl[1+(1-q)\, q^{k}\, x\, \frac{s_{n-1}(q^{k}\, x)}{\lambda _{n-1}(q^{k}\,x)} \biggr]^{-1}} $$

provided that

12 $$ \frac{s_{n}(x)}{\lambda _{n}(x)}=\frac{s_{n-1}(x)}{\lambda _{n-1}(x)},\quad n=1,2, \ldots , $$

where the functions $\lambda _{n}(x)$ and $s_{n}(x)$ are generated by

13 $$\begin{aligned}& \lambda _{n}(x) =D_{q}\, \lambda _{n-1}(x)+\lambda _{n-1}(q\, x)\, \lambda _{0}(x)+s_{n-1}(q\, x), \\& s_{n}(x) =D_{q}\,s_{n-1}(x)+\lambda _{n-1}(q\,x)\, s_{0}(x). \end{aligned}$$

For the proofs and detailed examples, we refer the reader to the recently published manuscript [26].

In the present work, we unify both the *d*AIM and *q*AIM using the Hahn operator (1). To achieve this goal, in Sect. 2 we summarize the relevant definitions and properties of the Hahn difference operator. Several novel properties of this operator are also introduced and proved in that section. In particular, the polynomials in the $(q;w)$-space are introduced, and the associated Taylor polynomial expansions are derived. In Sect. 3, the solutions of the first- and second-order linear Hahn difference equations

14 $$ \textstyle\begin{cases} D_{q;w} y(x)=\alpha (x)\, y(x)+\beta (x), \\ D_{q:w}^{2}\, y(x) = \lambda _{0}(x)\, D_{q:w} y(x) + s_{0}(x)\, y(x), \end{cases} $$

are examined. The necessary and sufficient conditions for the existence of polynomial solutions of the second-order linear Hahn difference equation are derived and proved. In Sect. 4, the solutions of the hypergeometric equation [28]

15 $$ \bigl(e\, x^{2}+2\, f\, x+g \bigr)\, D_{q;w}^{2}\, y(x)+(2 \,\varepsilon \, x+ \gamma )\, D_{q;w}\, y(x)+\tau \,y(x)=0 $$

are deduced. The necessary and sufficient conditions of the polynomial solutions are established and applied to construct the first few solutions explicitly.

## Preliminary definitions and properties of Hahn operator

To make this paper self-contained, we review the mathematical properties of the Hahn operator [1, 2, 10, 11, 18, 29–32] in this section and provide the proofs for other new properties developed for the present work.

### Theorem 1

*If* $f,g:I\to \mathbb{R}$ *and* $a,b\in \mathbb{R}$ *are* $q;w$-*differentiable functions and* $x\in I$, *then*:

1. *Linearity*:
  16 $$ D_{q;w} \bigl[a\, f(x) + b\, g(x) \bigr] = a \,D_{q;w}f(x) + b\, D_{q;w}g(x). $$
2. *Product rule*:
  17 $$\begin{aligned} D_{q;w} \bigl(f(x)\,g(x) \bigr) &= f(qx+w)D_{q;w}g(x) + g(x)D_{q;w}f(x) \\ &= g(qx+w)D_{q;w}f(x) + f(x)D_{q;w}g(x). \end{aligned}$$
3. *Quotient rule*:
  18 $$\begin{aligned} D_{q;w} \biggl(\frac{f(x)}{g(x)} \biggr) &= \frac{g(x)D_{q;w}f(x) - f(x)D_{q;w}g(x)}{g(qx+w)g(x)} \\ &=\frac{g(q\,x+w)D_{q;w}f(x) - f(q\,x+w)D_{q;w}g(x)}{g(qx+w)g(x)}, \end{aligned}$$
  *provided that* $g(qx+w)\,g(x)\neq 0$ *for all* $x\in I$.

### Lemma 1

*For* $q\ne 0$ *and* $n=1,2,\ldots $ ,

19 $$\begin{aligned} f \Biggl(q^{n} x+w\sum _{j=0}^{n-1} q^{j} \Biggr)&=f(x) \\ &\quad {}+ \bigl((q-1) x+w \bigr) \sum_{j=0}^{n-1} q^{j} D_{q;w} f \Biggl(q^{j}x+w \sum _{i=0}^{j-1}q^{i} \Biggr). \end{aligned}$$

### Proof

By definition

$$ \bigl[(q-1) x+w \bigr]D_{q;w} f ( x )=f(q x+w)-f(x) , $$

thus, replacing *x* with $q\,x+w$, it follows that

$$ q \bigl[(q-1) x+w \bigr]D_{q;w} f (q x+w ) =f \Biggl(q^{2} x+\sum_{j=0}^{1}q^{j} w \Biggr)-f(qx+ w). $$

Repeating the process, we have recursively that

20 $$\begin{aligned} &q^{k} \bigl[(q-1) x+w \bigr]D_{q;w} f \Biggl(q^{k}x+\sum _{j=0}^{k-1}q^{j}w \Biggr) \\ &\quad =f \Biggl(q^{k+1} x+\sum_{j=0}^{k} q^{j} w \Biggr)-f \Biggl(q^{k}x+\sum _{j=0}^{k-1} q^{j} \, w \Biggr). \end{aligned}$$

The assertion then follows by sum of these equations for $j=0$ to *k*. □

The following gives a generalization to Lemma 1.

### Lemma 2

*For* $x\neq w/(1-q)$,

21 $$\begin{aligned} &\sum_{j=0}^{n} q^{j} f \Biggl(q^{j}x+w\sum _{i=0}^{j-1}q^{i} \Biggr)D_{q;w} g \Biggl(q^{j}x+w\sum _{i=0}^{j-1}q^{i} \Biggr) \\ &\quad = \frac{f(q^{n+1} x+w\sum_{j=0}^{n} q^{j})\, g(q^{n+1} x+w\sum_{j=0}^{n} q^{j})-f(x)\, g(x)}{(q-1) x+w} \\ &\qquad {}-\sum_{j=0}^{n} q^{j} g \Biggl(q^{j}(qx+w)+w\sum_{i=0}^{j-1}q^{i} \Biggr)D_{q;w} f \Biggl(q^{j}x+w\sum _{i=0}^{j-1}q^{i} \Biggr), \end{aligned}$$

*where identity* (19) *follows for* $g(x)$ *or* $f(x)$ *is a constant function*.

### Proof

We note

$$\begin{aligned} &\sum_{j=0}^{n} q^{j} D_{q;w} \Biggl[ f \Biggl(q^{j}x+w\sum _{i=0}^{j-1}q^{i} \Biggr)\,g \Biggl(q^{j}x+w\sum_{i=0}^{j-1}q^{i} \Biggr) \Biggr] \\ &\quad = \frac{f(q^{n+1} x+w\sum_{j=0}^{n} q^{j})g(q^{n+1} x+w\sum_{j=0}^{n} q^{j})-f(x)g(x)}{(q-1) x+w}. \end{aligned}$$

By the product rule of $D_{q;w} [ f (q^{j}x+w\sum_{i=0}^{j-1}q^{i} )\,g (q^{j}x+w\sum_{i=0}^{j-1}q^{i} ) ]$, see (17), we have

$$\begin{aligned} &\sum_{j=0}^{n} q^{j} \Biggl[g \Biggl(q^{j}(qx+w)+w\sum_{i=0}^{j-1}q^{i} \Biggr)D_{q;w} f \Biggl(q^{j}x+w\sum _{i=0}^{j-1}q^{i} \Biggr) \\ &\qquad {}+ f \Biggl(q^{j}x+w\sum_{i=0}^{j-1}q^{i} \Biggr)D_{q;w} g \Biggl(q^{j}x+w\sum _{i=0}^{j-1}q^{i} \Biggr) \Biggr] \\ &\quad = \frac{f(q^{n+1} x+w\sum_{j=0}^{n} q^{j})g(q^{n+1} x+w\sum_{j=0}^{n} q^{j})-f(x)g(x)}{(q-1) x+w}, \end{aligned}$$

which completes the proof after some simple arrangements. □

### Theorem 2

*For a* $q;w$-*differentiable function* *f*,

22 $$ f \Biggl(q^{n}x+w\sum_{k=0}^{n-1}q^{k} \Biggr)=\sum_{k=0}^{n} { \binom{n}{k}}_{q}\, q^{\frac{1}{2} k(k-1)}\, \bigl[(q-1)\, x+w \bigr]^{k}\, D_{q;w}^{k} f(x), $$

*where* $\binom{n}{k}_{q}$ *is the* *q*-*binomial coefficients* [27, *formula* 12.1.4].

### Proof

We prove this by induction on *n*. The result holds for $n=1$ by the definition

$$ f (q\,x+w )=f(x)+ \bigl[(q-1)\, x+w \bigr] D_{q;w} f(x). $$

For the inductive step, assume that for some *n* the theorem is true. For $n+1$, it follows using the well-know identity [33], for $n\geq 1$ and $1\le k\le n$,

23 $$ {\binom{n+1}{k}}_{q}= { \binom{n}{k}}_{q}+ q^{n+1-k}{\binom{n}{k-1}}_{q}, $$

that

$$\begin{aligned} &\sum_{k=0}^{n+1} {\binom{n+1}{k}}_{q} q^{k(k-1)/2} \bigl[(q-1)\, x+w \bigr]^{k} D_{q;w}^{k} f(x) \\ &\quad =\sum_{k=0}^{n+1} { \binom{n}{k}}_{q} q^{k(k-1)/2} \bigl[(q-1)\, x+w \bigr]^{k} D_{q;w}^{k} f(x) \\ &\qquad {}+\sum_{k=0}^{n+1} q^{n+1-k}{ \binom{n}{k-1}}_{q} q^{k(k-1)/2} \bigl[(q-1) \, x+w \bigr]^{k} D_{q;w}^{k} f(x). \end{aligned}$$

However, using the properties of the *q*-binomial coefficient ${\binom{n}{n+1}}_{q}=0$ and ${\binom{n}{0}}_{q}=1$, we have

$$\begin{aligned} &\sum_{k=0}^{n+1} {\binom{n+1}{k}}_{q} q^{k(k-1)/2} \bigl[(q-1)\, x+w \bigr]^{k} D_{q;w}^{k} f(x) \\ &\quad =\sum_{k=0}^{n} {\binom{n}{k}}_{q} q^{k(k-1)/2} \bigl[(q-1)\, x+w \bigr]^{k} D_{q;w}^{k} f(x) \\ &\qquad {}+\sum_{k=1}^{n+1} q^{n+1-k}{ \binom{n}{k-1}}_{q} q^{k(k-1)/2} \bigl[(q-1) \, x+w \bigr]^{k} D_{q;w}^{k} f(x). \end{aligned}$$

For $1\le k\le n+1$, we have for $j=k-1$, where $0\leq j\le n$, that

$$\begin{aligned} &\sum_{k=0}^{n+1} {\binom{n+1}{k}}_{q} q^{k(k-1)/2} \bigl[(q-1)\, x+w \bigr]^{k} D_{q;w}^{k} f(x) \\ &\quad =\sum_{k=0}^{n} {\binom{n}{k}}_{q} q^{k(k-1)/2} \bigl[(q-1)\, x+w \bigr]^{k} D_{q;w}^{k} f(x) \\ &\qquad {}+\sum_{j=0}^{n} q^{n-j}{ \binom{n}{j}}_{q} q^{{(j+1)}j/2} \bigl[(q-1)\, x+w \bigr]^{j+1} D_{q;w}^{j+1} f(x) \\ &\quad =f \Biggl(q^{n} x+w\sum_{k=0}^{n-1}q^{k} \Biggr)+q^{n} \bigl[(q-1)\, x+w \bigr] \\ &\qquad {}\times \sum_{j=0}^{n}{ \binom{n}{j}}_{q} q^{{j(j-1)}/2} \bigl[(q-1)\, x+w \bigr]^{j} D_{q;w}^{j} \bigl(D_{q;w}f(x) \bigr), \end{aligned}$$

which, using (20), finally implies

$$\begin{aligned} &\sum_{k=0}^{n+1} { \binom{n+1}{k}}_{q} q^{k(k-1)/2} \bigl[(q-1)\, x+w \bigr]^{k} D_{q;w}^{k} f(x) \\ &\quad =f \Biggl(q^{n} x+w\sum_{k=0}^{n-1}q^{k} \Biggr)+q^{n} \bigl[(q-1)\, x+w \bigr]D_{q;w} f \Biggl(q^{n} x+w\sum_{k=0}^{n-1}q^{k} \Biggr) \\ &\quad = f \Biggl(q^{n+1} x+w\sum_{k=0}^{n}q^{k} \Biggr). \end{aligned}$$

This completes the proof. □

For example, if $n=3$, then this lemma implies

24 $$\begin{aligned} &f \Biggl(q^{3}\,x+w\sum _{i=0}^{2} q^{i} \Biggr) \\ &\quad =f(x)+ \bigl(1+q+q^{2} \bigr) \bigl((q-1)x+w \bigr)D_{q;w}f(x) \\ &\qquad {}+q \bigl(q^{2}+q+1 \bigr) \bigl((q-1) x+w \bigr)^{2}D_{q;w}^{2}f(x)+q^{3} \bigl((q-1)x+w \bigr)^{3}D_{q;w}^{3}f(x). \end{aligned}$$

A slightly different form of the following theorem was given in [34, Lemma 2.1]; however, we give here a direct proof based on the method of mathematical induction.

### Theorem 3

*Let* *f* *be a* $q;w$ *differentiable function on* *I*. *Then*, *for* $x\neq w/(1-q)$, *the* *nth* $q;w$ *derivative is evaluated using*

25 $$\begin{aligned} D_{q;w}^{n} f(x)&=\frac{q^{-n(n-1)/2}}{((q-1)\, x+w)^{n}} \\ &\quad {}\times \sum_{k=0}^{n} (-1)^{n-k}\, q^{(n-k) (n-k-1)/2}\, \binom{n}{k}_{q} \, f \Biggl(q^{k}\,x+w \sum_{i=0}^{k-1} q^{i} \Biggr). \end{aligned}$$

### Proof

Again, by induction on *n*, the result holds for $n=1$ by

$$ D_{q;w} f(x) = \bigl((q-1) x+w \bigr)^{-1} \bigl[f(q \,x+w)-f(x) \bigr]. $$

Assume it is true for *n*. Then, for $n+1$,

$$\begin{aligned} D_{q;w}^{n+1} f(x)&=D_{q;w}^{n} \bigl(D_{q;w} f(x) \bigr) \\ &= \frac{q^{-n(n-1)/2}}{((q-1) x+w)^{n}} \sum_{k=0}^{n} (-1)^{n-k} q^{(n-k) (n-k-1)/2} \binom{n}{k}_{q} D_{q;w} f \Biggl(q^{k}\,x+w \sum _{i=0}^{k-1} q^{i} \Biggr). \end{aligned}$$

However, by definition,

$$ D_{q;w} f \Biggl(q^{k}\,x+w \sum _{i=0}^{k-1} q^{i} \Biggr)= \frac{f(q^{k+1} x+w\sum_{j=0}^{k} q^{j} )-f(q^{k}x+w\sum_{j=0}^{k-1} q^{j})}{q^{k}[(q-1) x+w]}, $$

it follows that

$$\begin{aligned} D_{q;w}^{n+1} f(x)&=\frac{q^{-n(n-1)/2}}{((q-1) x+w)^{n+1}} \\ &\quad {}\times \sum_{k=0}^{n} (-1)^{n-k} q^{(n-k) (n-k-1)/2-k} \binom{n}{k}_{q} f \Biggl(q^{k+1}\,x+w \sum_{i=0}^{k} q^{i} \Biggr) \\ &\quad {}-\frac{q^{-n(n-1)/2}}{((q-1) x+w)^{n+1}} \\ &\quad {}\times \sum_{k=0}^{n} (-1)^{n-k} q^{(n-k) (n-k-1)/2-k} \binom{n}{k}_{q}f \Biggl(q^{k}\,x+w \sum_{i=0}^{k-1} q^{i} \Biggr). \end{aligned}$$

Shifting the index in the first sum, it follows that

$$\begin{aligned} D_{q;w}^{n+1} f(x)&={q^{-n(n-1)/2}} { \bigl((q-1) x+w \bigr)^{-n-1}} \\ &\quad {}\times \sum_{k=1}^{n+1} (-1)^{n-k+1} q^{(n-k+1) (n-k)/2-k+1} \binom{n}{k-1}_{q} f \Biggl(q^{k}\,x+w \sum_{i=0}^{k-1} q^{i} \Biggr) \\ &\quad {}-q^{-n(n-1)/2} \bigl((q-1) x+w \bigr)^{-n-1} \\ &\quad {}\times \sum_{k=0}^{n} (-1)^{n-k} q^{(n-k) (n-k-1)/2-k} \binom{n}{k}_{q}f \Biggl(q^{k}\,x+w \sum_{i=0}^{k-1} q^{i} \Biggr) \end{aligned}$$

and using ${\binom{n}{n+1}}_{q}= {\binom{n}{-1}}_{q}=0$, this expression can be written as

$$\begin{aligned} D_{q;w}^{n+1} f(x)&={q^{-n(n-1)/2}} { \bigl((q-1) x+w \bigr)^{-n-1}} \\ &\quad {}\times \sum_{k=0}^{n+1} (-1)^{n-k+1} q^{(n-k+1) (n-k)/2-k+1} \binom{n}{k-1}_{q} f \Biggl(q^{k}\,x+w \sum_{i=0}^{k-1} q^{i} \Biggr) \\ &\quad {}-{q^{-n(n-1)/2}} { \bigl((q-1) x+w \bigr)^{-n-1}} \\ &\quad {}\times \sum_{k=0}^{n+1} (-1)^{n-k} q^{(n-k) (n-k-1)/2-k} \binom{n}{k}_{q}f \Biggl(q^{k}\,x+w \sum_{i=0}^{k-1} q^{i} \Biggr). \end{aligned}$$

Further simplification yields

$$\begin{aligned} D_{q;w}^{n+1} f(x)&=\frac{q^{-n(n-1)/2}}{((q-1) x+w)^{n+1}} \sum _{k=0}^{n+1} (-1)^{n-k+1}q^{ (n-k-1)(n-k)/2-k} \\ &\quad {}\times \biggl[q^{n-k+1}\binom{n}{k-1}_{q}+ \binom{n}{k}_{q} \biggr]f \Biggl(q^{k}\,x+w \sum_{i=0}^{k-1} q^{i} \Biggr) \end{aligned}$$

or simply using (23)

$$\begin{aligned} D_{q;w}^{n+1} f(x)&= \frac{q^{-\frac{1}{2} n (n+1)}}{((q-1) x+w)^{n+1}} \\ &\quad {}\times \sum_{k=0}^{n+1} (-1)^{n-k+1}q^{\frac{1}{2} (n-k) (n+1-k)} \binom{n+1}{k}_{q} f \Biggl(q^{k}\,x+w \sum_{i=0}^{k-1} q^{i} \Biggr) \end{aligned}$$

as required. □

### Remark 1

For arbitrary $n=1,2,\ldots $ , the identity

26 $$ \sum_{k=0}^{n} (-1)^{k}\, q^{k\,(k-1)/2}\, \binom{n}{n-k}_{q}=0 $$

follows directly from Theorem 3 for the constant function $f(x)$ and the fact that $\sum_{k=0}^{n} A_{n-k}=\sum_{k=0}^{n} A_{k}$. Further, for $0< m< n$,

27 $$ \sum_{k=0}^{n} (-1)^{k} q^{\frac{1}{2} (n-k) (n-k-1)} \binom{n}{k}_{q} \Biggl(q^{k}\,x +w \sum_{i=0}^{k-1} q^{i} \Biggr)^{m}=0. $$

### Remark 2

For arbitrary $n,m=1,2,\ldots $ ,

28 $$\begin{aligned} &D_{q;w}^{n}\, x^{m} \\ &\quad = \textstyle\begin{cases} 0, &\mbox{if }m< n, \\ \frac{q^{-n(n-1)/2} }{((1-q) x-w)^{n}}\sum_{k=0}^{n} (-1)^{k} q^{\frac{1}{2} (n-k) (n-k-1)} \binom{n}{k}_{q} (q^{k}\,x+w \sum_{i=0}^{k-1} q^{i} )^{m}, &\mbox{if }m\geq n, \end{cases}\displaystyle \end{aligned}$$

that follows directly from Theorem 3 for the function $f(x)=x^{m}$.

### Definition 1

We define the *n*th-degree polynomials in the $(q;w)$-space as

29 $$ \mathcal{P}_{n}(x) = \prod _{j=0}^{n-1} \Biggl(x-w\sum _{i=0}^{j-1}q^{i} \Biggr) $$

recalling the empty product and sum: $\prod_{k=0}^{-1} a_{k} = 1 $ and $\sum_{k=0}^{-1} a_{k} = 0$.

The first few polynomials are given explicitly as follows:

$$ \textstyle\begin{cases} \mathcal{P}_{0}(x)=1, \\ \mathcal{P}_{1}(x)=x, \mathcal{P}_{2}(x)=x(x-w), \\ P_{3}(x)=x (x-w) (x-(q+1)\, w), \\ \mathcal{P}_{4}(x)=x (x-w) (x-(q+1)\, w) (x- (q^{2}+q+1 )\, w ), \\ \mathcal{P}_{5}(x)=x (x-w) (x-(q+1)\, w) (x- (q^{2}+q+1 )\, w ) (x- (q^{3}+q^{2}+q+1 )\, w ). \end{cases} $$

### Theorem 4

*The* $q;w$-*derivative of the* *nth*-*degree polynomial* $\mathcal{P}_{n}(x)$ *is given by*

30 $$ \mathcal{P}_{0}(x)=1,\qquad D_{q;w} \mathcal{P}_{n}(x) = \Biggl( \sum_{j=0}^{n-1} q^{j} \Biggr)\, \mathcal{P}_{n-1}(x),\quad n\geq 1. $$

### Proof

For arbitrary *n*,

$$\begin{aligned} D_{q;w} \mathcal{P}_{n}(x)&= \frac{\prod_{j=0}^{n-1} (qx+w-w \sum_{i=0}^{j-1} q^{i} )-\prod_{j=0}^{n-1} (x-w \sum_{i=0}^{j-1} q^{i} )}{(q-1)x+w} \\ &= \frac{q^{n} ( x- \frac{w (q^{-1}-1 )}{q-1} )\prod_{j=1}^{n-1} ( x- \frac{w (q^{j-1}-1 )}{q-1} )-\prod_{j=0}^{n-1} (x-\frac{w (q^{j}-1 )}{q-1} )}{(q-1)x+w}. \end{aligned}$$

Since $1\le j\le n-1$ implies $0\le j-1\le n-2$, let $i=j-1$, and it follows that $j=i+1$. Thus

$$\begin{aligned} D_{q;w} \mathcal{P}_{n}(x)&= \frac{q^{n} ( x- \frac{w (q^{-1}-1 )}{q-1} )\prod_{j=0}^{n-2} ( x- \frac{w (q^{j}-1 )}{q-1} )-\prod_{j=0}^{n-1} (x-\frac{w (q^{j}-1 )}{q-1} )}{(q-1)x+w} \\ &=\prod_{j=0}^{n-2} \biggl( x- \frac{w (q^{j}-1 )}{q-1} \biggr) \frac{q^{n} ( x- \frac{w (q^{-1}-1 )}{q-1} )- (x-\frac{w (q^{n-1}-1 )}{q-1} )}{(q-1)x+w} \\ &=\prod_{j=0}^{n-2} \biggl( x- \frac{w (q^{j}-1 )}{q-1} \biggr)\frac{q^{n}-1}{q-1}= \Biggl(\sum _{j=0}^{n-1} q^{j} \Biggr) \prod _{j=0}^{n-2} \biggl( x- \frac{w (q^{j}-1 )}{q-1} \biggr) \\ &= \Biggl(\sum_{j=0}^{n-1} q^{j} \Biggr) \mathcal{P}_{n-1}(x) \end{aligned}$$

as required. □

### Theorem 5

*In terms of the polynomials* $\{ \mathcal{P}_{n}(x)\}_{n=0}$, *an arbitrary* *r* *degree polynomial* $P_{r}(x)$ *can be represented by the following expansion*:

31 $$ P_{r}(x)=\sum_{k=0}^{r} \frac{D_{q;w}^{k} P_{r}(0)}{(1-q)^{-k}(q;q)_{k}} \mathcal{P}_{k}(x). $$

### Proof

Let $P_{r}(x)$ be the $r^{th}$ degree polynomial of *x* that can be written as

$$ P_{r}(x)=\sum_{k=0}^{r}A_{k} \, \mathcal{P}_{k}(x), $$

where $A_{k}$ are constants to be determined. Using the direct derivative for $k=0,1,2,\ldots $ , followed by the substitution $x=0$ to compute the corresponding $A_{k}$, it is not difficult to prove that

$$\begin{aligned} A_{k}&= \frac{D_{q;w}^{k} P_{r}(0)}{(1+q)(1+q+q^{2})(1+q+q^{2}+q^{3})\cdots (1+q+q^{2}+\cdots +q^{k-1})} \\ &= \frac{D_{q;w}^{k} P_{r}(0)}{\prod_{j=0}^{k-1} (\sum_{i=0}^{j} q^{i} )}= \frac{D_{q;w}^{k} P_{r}(0)}{(1-q)^{-k}(q;q)_{k}}. \end{aligned}$$

Thus,

$$ P_{r}(x)=\sum_{k=0}^{r} \frac{D_{q;w}^{k} P_{r}(0)}{(1-q)^{-k}(q;q)_{k}} \Biggl(\prod_{j=0}^{k-1} \Biggl(x-w\sum_{i=0}^{j-1}q^{j} \Biggr) \Biggr). $$

 □

For example, the expansions of the polynomials $x^{2}$, $x^{3}$, $x^{4}$ in terms of $\{\mathcal{P}_{n}(x)\}$ are

32 $$\begin{aligned} &x^{2}= w\, x + x\, (x - w), \\ &x^{3}=w^{2}\,x +(q\,w+2w)\,x\,(x-w)+x (x-w) \bigl(x-(q+1) w \bigr), \\ &\begin{aligned}[b] x^{4}&=w^{3}\,x+ \bigl(q^{2}+3 q+3 \bigr) \, w^{2}\, x\, (x-w) \\ &\quad {}+ \bigl(q^{2}+2 q+3 \bigr)\, w\, x\, (x-w)\, \bigl(x-(1+q) w \bigr) \\ &\quad {}+ x\, (x-w) \, \bigl(x-(1+q)\, w \bigr)\, \bigl(x- \bigl(1+q+q^{2} \bigr)\, w \bigr). \end{aligned} \end{aligned}$$

### Corollary 1

*If* $P_{r}(x)$ *is an* *r*-*degree polynomial*, *then*

$$ D_{q;w}^{n} P_{r}(x)=0,\quad \textit{for all }n\geq r+1. $$

## ${}_{q;w}\mbox{AIM}$ for Hahn difference equations

Several papers over the past few years have addressed the theory of the linear Hahn difference equations [17, 35–40]. The existence and uniqueness theorems of solutions of linear Hahn difference equations are given in [35]. In [10], Annaby, Hamza, and Aldwoah solved the first order linear Hahn difference equations with constant coefficients.

### First-order linear Hahn difference equations

We consider in this subsection the first-order Hahn difference equation with variable coefficients

33 $$ D_{q;w} y(x)=\alpha (x)\, y(x)+\beta (x), \quad 0< w, 0< q< 1, $$

where $\alpha (x)$ and $\beta (x)$ are arbitrary functions. Note that, using Hahn’s operator definition (1), equation (33) can be written as

34 $$ y(q\,x+w)= \bigl(1+ \bigl((q-1)\,x+w \bigr)\,\alpha (x) \bigr) \, y(x)+ \bigl((q-1)\,x+w \bigr)\,\beta (x) . $$

#### Theorem 6

*For* $0< q<1$, *the general solution of the* $q;w$-*difference equation of the first*-*order Hahn difference equation* (33) *or* (34) *is*

35 $$\begin{aligned} y(x)&= \frac{c}{\prod_{k=0}^{\infty } (1+q^{k} ((q-1) x+w)\, \alpha (w \sum_{i=0}^{k-1} q^{i}+x\, q^{k} ) )} \\ &\quad {}-\sum_{k=0}^{\infty } \frac{q^{k} ((q-1) x+w)\, \beta (w \sum_{i=0}^{k-1} q^{i}+x q^{k} )}{\prod_{j=0}^{k} (1+q^{j} ((q-1) x+w)\, \alpha (w \sum_{i=0}^{j-1} q^{i}+x\, q^{j} ) )}, \end{aligned}$$

*where* $c(qx+w)=c(x)$ *is a periodic* (*constant*) *function*.

#### Proof

We prove this theorem by direct substitution. By the given solution

$$\begin{aligned} y(q x+w) &= \frac{~c~}{\prod_{k=0}^{\infty } (1+q^{k+1} ((q-1)x+w) \alpha (w \sum_{i=0}^{k} q^{i}+q^{k+1}x )} \\ &\quad {}-\sum_{k=0}^{\infty } \frac{q^{k+1} ((q-1) x+w) \beta (w \sum_{i=0}^{k} q^{i}+q^{k+1}x )}{\prod_{j=0}^{k} (1+q^{j+1} ((q-1)x+w) \alpha (w \sum_{i=0}^{j} q^{i}+ q^{j+1}x ) )}, \end{aligned}$$

shift the indices of the finite products

$$\begin{aligned} y(q x+w)&= \frac{~c~}{\prod_{k=1}^{\infty } (1+q^{k} ((q-1)x+w) \alpha (w \sum_{i=0}^{k-1} q^{i}+q^{k}x )} \\ &\quad {}-\sum_{k=1}^{\infty } \frac{q^{k} ((q-1) x+w) \beta (w \sum_{i=0}^{k-1} q^{i}+q^{k}x )}{\prod_{j=0}^{k-1} (1+q^{j+1} ((q-1)x+w) \alpha (w \sum_{i=0}^{j} q^{i}+ q^{j+1}x ) )}, \end{aligned}$$

which can be written as

$$\begin{aligned} y(q x+w)&= \frac{(1+((q-1)x+w) \alpha (x))~c~}{\prod_{k=0}^{\infty } (1+q^{k} ((q-1)x+w) \alpha (w \sum_{i=0}^{k-1} q^{i}+q^{k}x )} \\ &\quad {}-\sum_{k=1}^{\infty } \frac{q^{k} ((q-1) x+w) \beta (w \sum_{i=0}^{k-1} q^{i}+q^{k}x )}{\prod_{j=0}^{k-1} (1+q^{j+1} ((q-1)x+w) \alpha (w \sum_{i=0}^{j} q^{i}+ q^{j+1}x ) )}. \end{aligned}$$

Using the given solution again, it follows that

$$\begin{aligned} y(q x+w)&= \bigl(1+ \bigl((q-1)x+w \bigr) \alpha (x) \bigr) \\ &\quad {}\times \Biggl(y(x)+\sum_{k=0}^{\infty } \frac{q^{k} ((q-1) x+w) \beta (w \sum_{i=0}^{k-1} q^{i}+q^{k}\,x )}{\prod_{j=0}^{k} (q^{j} ((q-1) x+w) \alpha (w \sum_{i=0}^{j-1} q^{i}+x q^{j} )+1 )} \Biggr) \\ &\quad {}-\sum_{k=1}^{\infty } \frac{q^{k} ((q-1) x+w)\, \beta (w \sum_{i=0}^{k-1} q^{i}+q^{k}x )}{\prod_{j=0}^{k-1} (1+q^{j+1} ((q-1)x+w)\, \alpha (w \sum_{i=0}^{j} q^{i}+ q^{j+1}x ) )}, \end{aligned}$$

which simplifies to

$$ y(q\, x+w)= \bigl(1+ \bigl((q-1)x+w \bigr)\, \alpha (x) \bigr)\, y(x)+ \bigl((q-1) x+w \bigr)\,\beta (x). $$

 □

#### Remark 3

For $0< q<1$, a solution of the homogeneous $q;w$-difference equation

$$ D_{q;w} y(x)= \alpha (x) \, y (x) $$

up to a multiplicative constant is

36 $$ y(x) ={\prod_{k=0}^{\infty } \Biggl(1+q^{k} \bigl((q-1) x+w \bigr)\, \alpha \Biggl(w \sum _{i=0}^{k-1} q^{i}+x\, q^{k} \Biggr) \Biggr)^{-1}}. $$

#### Remark 4

The exact solution of the limiting case $w\to 0$ [26]

$$ D_{q} y(x)=\alpha (x)\, y(x)+\beta (x),\quad 0< q< 1, $$

is

37 $$ y(x)= \frac{c}{\prod_{k=0}^{\infty } (1-q^{k} (1-q)\,x\, \alpha (q^{k}\,x ) )}- \sum_{k=0}^{\infty } \frac{q^{k} (q-1)\, x\, \beta ( q^{k}\,x )}{\prod_{j=0}^{k} (1+q^{j} (q-1) x\, \alpha (q^{j}\,x ) )} $$

as developed earlier in [26].

#### Example 1

The solution of the first-order Hahn difference equation

$$ D_{q;w}\, y(x)=y(x),\quad 0< q< 1, $$

up to some multiplicative constant is

$$ y(x)= \frac{1}{\prod_{k=0}^{\infty } (1+q^{k} ((q-1) x+w) )}= \frac{1}{((1-q)\,x -w;q)_{\infty }}, $$

which is the $q;w$-exponential function [27, Theorem 12.2.6] as expected for this difference equation.

#### Example 2

The solution of the first-order Hahn difference equation

$$ D_{q;w}\, y(x)=a\, y(x)+b, \quad 0< q< 1, $$

where *a* and *b* are real constants, is

$$ y(x)=\frac{c}{(a ((1-q) x-w);q)_{\infty }}-\sum_{k=0}^{\infty } \frac{b\, q^{k} ((q-1) x+w)}{(a ((1-q) x-w);q)_{k+1}}. $$

### ${}_{q;w}\mbox{AIM}$ for the second-order Hahn difference equations

In this section, we consider the second-order Hahn difference equation

38 $$ y \bigl(q^{2}\,x+(1+q)\, w \bigr)=\alpha (x) \, y(q \, x+w)+\beta (x)\, y(x),$$

where $\alpha (x)$ and $\beta (x)$ are $(q;w)$-differentiable functions. Using the easily proved identity

39 $$ D_{q;w}^{2}f(x)= \frac{f(q^{2}\,x+(1+q)w)-(1+q)f(qx+w) +qf(x)}{q((q-1)x+w)^{2}}, $$

equation (38) can be written equivalently as

40 $$ D_{q;w}^{2} y(x)=\lambda _{0}(x)\, D_{q;w} y(x)+s_{0}(x)\, y(x), $$

where

41 $$ \lambda _{0}(x)=\frac{\alpha (x)-q-1}{q\, ((q-1)\, x+w)},\qquad s_{0}(x)= \frac{\alpha (x)+\beta (x)-1}{q\, ((q-1)\,x+w)^{2}}. $$

Although these two forms (38) and (40) are equivalent, we shall focus our attention on the form (40) to investigate the solutions of the second-order linear Hahn difference equation (40) with variable coefficients.

#### General solution

Acting by the Hahn difference operator $D_{q;w}$ on equation (40) yields

$$ D_{q;w}^{3}\, y(x) =D_{q;w} \bigl(D_{q;w}^{2}\, y(x) \bigr)= D_{q;w} \bigl( \lambda _{0}(x)\, D_{q;w}\, y(x) \bigr)+D_{q;w} \bigl(s_{0}(x) \, y(x) \bigr). $$

However, by means of the product rule (17), it easily follows that

$$ D_{q;w}^{3}\, y(x)=\lambda _{1}(x)D_{q;w} y(x)+s_{1}(x)y(x), $$

where

$$ \textstyle\begin{cases} \lambda _{1}(x)= D_{q;w}\lambda _{0}(x)+\lambda _{0}(qx+w)\lambda _{0}(x)+s_{0}(qx+w), \\ s_{1}(x)= D_{q;w}s_{0}(x)+\lambda _{0}(qx+w)s_{0}(x). \end{cases} $$

Similarly, applying the Hahn difference once more yields

$$ D_{q;w}^{4}\, y(x)=\lambda _{2}(x) D_{q;w}y(x)+s_{2}(x)\, y(x), $$

where

$$ \textstyle\begin{cases} \lambda _{2}(x)= D_{q;w}\,\lambda _{1}(x)+\lambda _{1}(qx+w)\, \lambda _{0}(x)+s_{1}(qx+w), \\ s_{2}(x)=D_{q;w}\,s_{1}(x)+\lambda _{1}(qx+w)\,s_{0}(x). \end{cases} $$

In general, we may apply this process to obtain, for $n=1,2,\ldots $ , that

42 $$\begin{aligned}& D_{q;w}^{n+1}\, y(x) =\lambda _{n-1}(x)\, D_{q;w}\, y(x)+s_{n-1}(x)\, y(x), \end{aligned}$$

43 $$\begin{aligned}& D_{q;w}^{n+2}\, y(x) =\lambda _{n}(x)\, D_{q;w}\, y(x)+s_{n}(x)\, y(x), \end{aligned}$$

where

44 $$ \textstyle\begin{cases} \lambda _{n}(x)= D_{q;w}\,\lambda _{n-1}(x)+\lambda _{n-1}(q\,x+w)\, \lambda _{0}(x)+s_{n-1}(q\, x+w), \\ s_{n}(x)= D_{q;w}\,s_{n-1}(x)+\lambda _{n-1}(q\,x+w)\,s_{0}(x). \end{cases} $$

The general form (43) can be proved by induction on *n*. For $n=1$, the identity is true by construction. Assume it is true for $n+1$, then for $n+2$

$$\begin{aligned} D_{q;w}^{n+2}\, y(x)&=D_{q;w} \bigl(D_{q;w}^{n+1}y(x) \bigr) \\ &=D_{q;w} \bigl(\lambda _{n-1}(x)\, D_{q;w}\, y(x)+s_{n-1}(x)\, y(x) \bigr), \end{aligned}$$

and by the product rule (17),

$$\begin{aligned} D_{q;w}^{n+2}\, y(x)&=\lambda _{n-1}(qx+w)\, D_{q;w}^{2}\, y(x)+D_{q;w} y(x)D_{q;w} \lambda _{n-1}(x) \\ &\quad {}+s_{n-1}(qx+w)D_{q;w} y(x)+y(x)D_{q;w} s_{n-1}(x). \end{aligned}$$

Using (40) and simplifying, we obtain

$$\begin{aligned} D_{q;w}^{n+2}\, y(x)&= \bigl(D_{q;w}\lambda _{n-1}(x) +\lambda _{n-1}(qx+w) \lambda _{0}(x)+s_{n-1}(qx+w) \bigr)D_{q;w}y(x) \\ &\quad {}+ \bigl(D_{q;w}s_{n-1}(x)+ \lambda _{n-1}(x)s_{0}(x) \bigr)y(x) \\ &=\lambda _{n}(x)D_{q;w}y(x)+s_{n}(x)y(x) \end{aligned}$$

as expected. Now dividing (42) and (41), we obtain

$$ \frac{D_{q;w}^{n+2}\, y(x)}{D_{q;w}^{n+1}\, y(x)}= \frac{\lambda _{n}(x) [D_{q;w}y(x)+\frac{s_{n}(x)}{\lambda _{n}(x) } y(x) ]}{\lambda _{n-1}(x) [D_{q;w}y(x)+\frac{s_{n-1}(x)}{\lambda _{n-1}(x)} y(x) ]}. $$

Thus, if for some $n\geq 1$ the so-called terminating condition

45 $$ \frac{s_{n}(x)}{\lambda _{n}(x) } = \frac{s_{n-1}(x)}{\lambda _{n-1}(x) } $$

is satisfied, then it follows that

46 $$ \frac{D_{q;w}^{n+2}\, y(x)}{D_{q;w}^{n+1}\, y(x)}= \frac{\lambda _{n}(x)}{\lambda _{n-1}(x)}. $$

This is easily written as a first-order difference equation in $D_{q;w}^{n+1}\, y(x)$

47 $$ D_{q;w} \bigl[D_{q;w}^{n+1}\, y(x) \bigr]= \frac{\lambda _{n}(x)}{\lambda _{n-1}(x)} {D_{q;w}^{n+1}\, y(x)} $$

with a solution for $0< q<1$ given by, see Theorem 6,

48 $$ D_{q;w}^{n+1} y(x) = \frac{c_{2}}{\prod_{k=0}^{\infty } [1+q^{k}({w+(q-1)\,x})\,\frac{\lambda _{n}(q^{k}x+w\sum_{i=0}^{k-1}q^{i})}{\lambda _{n-1}(q^{k}x+w\sum_{i=0}^{k-1}q^{i})} ]} $$

for some periodic (constant) function $c_{2}$. Equation (41) reduces to

$$\begin{aligned} D_{q;w}y(x)&= -\frac{s_{n-1}(x)}{\lambda _{n-1}(x) }\, y(x) \\ &\quad {}+ \frac{1}{\lambda _{n-1}(x) } \frac{c_{2}}{\prod_{i=0}^{\infty } [1+q^{i}({w+(q-1)\,x})\frac{\lambda _{n}(q^{i}\, x+w\sum_{l=0}^{i-1}q^{l})}{\lambda _{n-1}(q^{i}x+w\sum_{l=0}^{i-1}q^{l})} ]}, \end{aligned}$$

with the general solution given by

49 $$\begin{aligned} y(x) &= \frac{c_{1}}{\prod_{k=0}^{\infty } [1+q^{k}({(1-q)\,x-w})\,\frac{s_{n-1}(x_{k})}{\lambda _{n-1}(x_{k})} ]} \\ &\quad {}-\sum_{i=0}^{\infty } \frac{\frac{q^{i}((q-1)x+w)}{\lambda _{n-1}(x_{i})}}{\prod_{j=0}^{i} [1+q^{j}((1-q)x-w)\frac{s_{n-1}(x_{j})}{\lambda _{n-1}(x_{j})}] } \\ &\quad {}\times \frac{c_{2}}{\prod_{j=0}^{\infty } [1+q^{i+j} ((q-1) x+w) \frac{\lambda _{n}(q^{i+j}\,x+w \sum_{l=0}^{i+j-1} q^{l})}{\lambda _{n-1}(q^{i+j}\,x+w \sum_{l=0}^{i+j-1} q^{l})} ]}, \end{aligned}$$

where $x_{k}=q^{k}\,x+w\sum_{j=0}^{k-1} q^{j}$ and $c_{i}$, $i=1,2$, are periodic functions with the property that $D_{q;w}\, c_{i}=0$, $i=1,2$. It is a straightforward exercise to show that the two solutions are linearly independent by proving that the $(q;w)$*-Casorati determinant*

50 $$ \begin{vmatrix} y_{1}(x) & y_{1}(q\,x+w) \\ y_{2}(x) & y_{2}(q\, x+w) \end{vmatrix} $$

does not vanish. We summarize this result in the following theorem.

##### Theorem 7

*A solution of the second*-*order Hahn linear difference equation*

$$ D_{q:w}^{2} y(x) = \lambda _{0}(x)\, D_{q:w} y(x) + s_{0}(x)\, y(x) $$

*is given by*

$$ y_{n}(x)={\prod_{k=0}^{\infty } \biggl[1+q^{k} \bigl({(1-q)\,x-w} \bigr)\, \frac{s_{n-1}(x_{k})}{\lambda _{n-1}(x_{k})} \biggr]^{-1}} $$

*provided that*, *for some* $n>0$,

$$ \frac{s_{n}(x)}{\lambda _{n}(x) } = \frac{s_{n-1}(x)}{\lambda _{n-1}(x) } \quad \textit{or}\quad \delta _{n}(x) \equiv \lambda _{n}(x)s_{n-1}(x)- \lambda _{n-1}(x)s_{n}(x)=0, $$

*where*

$$ \textstyle\begin{cases} \lambda _{n}(x)= D_{q;w}\,\lambda _{n-1}(x)+\lambda _{n-1}(q\, x+w)\, \lambda _{0}(x)+s_{n-1}(q\, x+w), \\ s_{n}(x)= D_{q;w}\,s_{n-1}(x)+\lambda _{n-1}(q\,x+w)\,s_{0}(x). \end{cases} $$

##### Remark 5

In general, the terminating condition (45) is satisfied for $n\to \infty $. As discussed and proved in the next section, the condition holds for finite *n* if the difference equation can be solved exactly by a polynomial of at most *n* degree. As far as we know, it is not possible to predict whether such a condition will hold for specific *n* without an experiment.

#### A criterion for polynomial solutions

We give in Theorems 8 and 9 below the necessary and sufficient conditions respectively for a second order linear Hahn difference equation (40) to have a polynomial solution.

##### Theorem 8

*If a solution of the difference equation*

$$ D_{q:w}^{2}\, y(x) = \lambda _{0}(x)\, D_{q:w} y(x) + s_{0}(x)\, y(x) $$

*is a polynomial of at most of degree* *n*, *then* $\delta _{n}(x)=0$.

##### Proof

Multiplying equation (41) by $\lambda _{n}(x)$ and (42) by $\lambda _{n-1}(x)$ yields

$$\begin{aligned}& \lambda _{n}(x)D_{q;w}^{n+1}\, y(x) =\lambda _{n}(x)\lambda _{n-1}(x) D_{q;w}y(x)+ \lambda _{n}(x)s_{n-1}(x) y(x), \\& \lambda _{n-1}(x)D_{q;w}^{n+2}\, y(x) =\lambda _{n-1}(x)\lambda _{n}(x) D_{q;w}y(x)+\lambda _{n-1}(x)s_{n}(x) y(x). \end{aligned}$$

Subtracting these two equations, it follows that

$$\begin{aligned} \lambda _{n}(x)D_{q;w}^{n+1}y(x)-\lambda _{n-1}(x)D_{q;w}^{n+2}\, y(x)&= \bigl[ \lambda _{n}(x)s_{n-1}(x)-\lambda _{n-1}(x)s_{n}(x) \bigr] y(x) \\ &=\delta _{n}(x)y(x). \end{aligned}$$

Consequently, if $y(x)$ is a polynomial of at most *n*, it follows by Corollary 1 that $D_{q;w}^{n+2}y(x)=D_{q;w}^{n+1}y(x)=0$; and $\delta _{n}(x)=0$. □

##### Lemma 3

*If*

$$ y(x)= \frac{1}{\prod_{k=0}^{\infty } [1+q^{k}({(1-q)\,x-w})\,\frac{s_{n-1}(q^{k}x+w\sum_{i=0}^{k-1}q^{i})}{\lambda _{n-1}(q^{k}x+w\sum_{i=0}^{k-1}q^{i})} ]}, $$

*then*

51 $$ D_{q;w}y(x)=- \frac{s_{n-1}(x)}{\lambda _{n-1}(x)}\times y(x). $$

##### Proof

It is not difficult to show, by shifting the indices of the product definitions of $y(x)$ and $y(q\,x+w)$, that the difference $y(q\,x+w)-y(x)$ reduces to

$$\begin{aligned} y(qx+w)-y(x)&={\prod_{k=1}^{\infty } \biggl[1+q^{k} \bigl({(1-q) x-w} \bigr)\, \frac{s_{n-1}(q^{k}x+w\sum_{i=0}^{k-1}q^{i})}{\lambda _{n-1}(q^{k}x+w\sum_{i=0}^{k-1}q^{i})} \biggr]^{-1}} \\ &\quad {}- {\prod_{k=0}^{\infty } \biggl[1+q^{k} \bigl({(1-q)\,x-w} \bigr)\, \frac{s_{n-1}(q^{k}x+w\sum_{i=0}^{k-1}q^{i})}{\lambda _{n-1}(q^{k}x+w\sum_{i=0}^{k-1}q^{i})} \biggr]^{-1}}. \end{aligned}$$

Thus, by definition of the difference operator,

$$\begin{aligned} D_{q;w}y(x)&=\frac{y(qx+w)-y(x)}{(q-1)x+w} \\ &=y(x)\times \frac{{ [1+({(1-q) x-w})\,\frac{s_{n-1}(x)}{\lambda _{n-1}(x)} ]}- 1}{(q-1)x+w}=-y(x) \times \frac{s_{n-1}(x)}{\lambda _{n-1}(x)}. \end{aligned}$$

 □

##### Theorem 9

*If* $\delta _{n}=0$ *and* $\lambda _{n-1}(x)\neq 0$, *then the solution of the difference equation*

$$ D_{q:w}^{2}\, y(x) = \lambda _{0}(x)\, D_{q:w}\, y(x) + s_{0}(x)\, y(x) $$

*is a polynomial of at most degree* *n*.

##### Proof

Given $\delta _{n}(x)=0$, it follows by Theorem 6 that

$$ y(x)= \frac{1}{\prod_{k=0}^{\infty } [1+q^{k}({(1-q)\,x-w})\,\frac{s_{n-1}(q^{k}x+w\sum_{i=0}^{k-1}q^{i})}{\lambda _{n-1}(q^{k}x+w\sum_{i=0}^{k-1}q^{i})} ]}, $$

which allows one to evaluate $D_{q;w} y(x)$ as, see Lemma 3,

$$ D_{q;w}y(x)=-y(x)\times \frac{s_{n-1}(x)}{\lambda _{n-1}(x)}. $$

Thus, using equation (41), we have

$$\begin{aligned} D_{q;w}^{n+1}\, y(x)&=\lambda _{n-1}(x) D_{q;w}y(x)+s_{n-1}(x) y(x) \\ &= \biggl(-\lambda _{n-1}(x) \frac{s_{n-1}(x)}{\lambda _{n-1}(x)}+s_{n-1}(x) \biggr)y(x)=0. \end{aligned}$$

 □

##### Theorem 10

*Let* $\lambda _{n}(x)$ *and* $s_{n}(x)$ *be as in* (44), *and set* $\delta _{n}(x) = \lambda _{n}(x)\,s_{n-1}(x)-\lambda _{n-1}(x)\,s_{n}(x)$. *If* $\delta _{n}(x)=0$, *then* $\delta _{n'}(x)=0$ *for all* $n' \ge n+1$.

##### Proof

Note that, by the sequence relations of $\lambda _{n}(x)$ and $s_{n}(x)$, we have

$$\begin{aligned} \delta _{n+1}(x)&=\lambda _{n+1}(x)s_{n}(x)- \lambda _{n}(x)s_{n+1}(x) \\ &=s_{n}(x)D_{q;w}\,\lambda _{n}(x)+s_{n}(x) \lambda _{n}(qx+w)\, \lambda _{0}(x)+s_{n}(qx+w)s_{n}(x) \\ &\quad {}-\lambda _{n}(x)D_{q;w}\,s_{n}(x)-\lambda _{n}(x)\lambda _{n}(qx+w) \,s_{0}(x). \end{aligned}$$

This equation can be easily written as

$$\begin{aligned} \delta _{n+1}(x)&=s_{n}(qx+w)s_{n}(x) \biggl( \frac{s_{n}(x)D_{q;w}\,\lambda _{n}(x)-\lambda _{n}(x)D_{q;w}\,s_{n}(x)}{s_{n}(qx+w)s_{n}(x)} \biggr) \\ &\quad {}+s_{n}(qx+w)s_{n}(x)+s_{n}(x)\lambda _{n}(qx+w)\,\lambda _{0}(x)- \lambda _{n}(x) \lambda _{n}(qx+w)\,s_{0}(x), \end{aligned}$$

where the quotient rule yields

$$\begin{aligned} \delta _{n+1}(x)&=s_{n}(qx+w)s_{n}(x)D_{q;w} \biggl( \frac{\lambda _{n}(x)}{s_{n}(x)} \biggr) \\ &\quad {}+s_{n}(qx+w)s_{n}(x)+s_{n}(x)\lambda _{n}(qx+w)\,\lambda _{0}(x)- \lambda _{n}(x) \lambda _{n}(qx+w)\,s_{0}(x). \end{aligned}$$

Further simplification implies

$$\begin{aligned} \delta _{n+1}(x)&=s_{n}(qx+w)s_{n}(x) \\ &\quad {}\times \biggl[D_{q;w} \biggl(\frac{\lambda _{n}(x)}{s_{n}(x)} \biggr)+1+ \frac{\lambda _{n}(qx+w)}{s_{n}(qx+w)}\,\lambda _{0}(x)- \frac{\lambda _{n}(x)\lambda _{n}(qx+w)\,s_{0}(x)}{s_{n}(x)s_{n}(qx+w)} \biggr]. \end{aligned}$$

Using the terminating condition, it follows that

$$\begin{aligned} \delta _{n+1}(x)& =s_{n}(qx+w)s_{n}(x) \\ &\quad {}\times \biggl[D_{q;w} \biggl(\frac{\lambda _{n-1}(x)}{s_{n-1}(x)} \biggr)+1+ \frac{\lambda _{n-1}(qx+w)}{s_{n-1}(qx+w)}\,\lambda _{0}(x)- \frac{\lambda _{n-1}(x)\lambda _{n-1}(qx+w)\,s_{0}(x)}{s_{n-1}(x)s_{n-1}(qx+w)} \biggr], \end{aligned}$$

which implies

$$\begin{aligned} \delta _{n+1}(x)&=s_{n}(qx+w)s_{n}(x) \biggl[ \frac{s_{n-1}D_{q;w}\lambda _{n-1}(x)-\lambda _{n-1}(x)D_{n-1}s_{n-1}(x)}{s_{n-1}(x)s_{n-1}(qx+w)} \\ &\quad {}+1+\frac{\lambda _{n-1}(qx+w)}{s_{n-1}(qx+w)}\,\lambda _{0}(x)- \frac{\lambda _{n-1}(x)\lambda _{n-1}(qx+w)\,s_{0}(x)}{s_{n-1}(x)s_{n-1}(qx+w)} \biggr] \\ &=s_{n}(qx+w)s_{n}(x) \biggl[ \frac{s_{n-1}(x)D_{q;w}\lambda _{n-1}(x)-\lambda _{n-1}(x)D_{q;w}s_{n-1}(x)}{s_{n-1}(x)s_{n-1}(qx+w)} \\ &\quad {}+\frac{s_{n-1}(x)s_{n-1}(qx+w)}{s_{n-1}(x)s_{n-1}(qx+w)}+ \frac{s_{n-1}(x)\lambda _{n-1}(qx+w)}{s_{n-1}(x)s_{n-1}(qx+w)}\, \lambda _{0}(x) \\ &\quad {}- \frac{\lambda _{n-1}(x)\lambda _{n-1}(qx+w)\,s_{0}(x)}{s_{n-1}(x)s_{n-1}(qx+w)} \biggr]. \end{aligned}$$

Finally, we have

$$\begin{aligned} \delta _{n+1}(x) &=s_{n}(qx+w)s_{n}(x) \\ &\quad {}\times \biggl[ \frac{s_{n-1}(x) (D_{q;w}\lambda _{n-1}(x)+\lambda _{n-1}(qx+w)\lambda _{0}(x)+\lambda _{n-1}(qx+w) )}{s_{n-1}(x)s_{n-1}(qx+w)} \\ &\quad {} - \frac{\lambda _{n-1}(x) (D_{n-1}s_{n-1}(x)+\lambda _{n-1}(qx+w)\,s_{0}(x) )}{s_{n-1}(x)s_{n-1}(qx+w)} \biggr] \\ &=s_{n}(qx+w)s_{n}(x) \biggl[ \frac{s_{n-1}(x)\lambda _{n}(x)-\lambda _{n-1}(x)s_{n}(x)}{s_{n-1}(x)s_{n-1}(qx+w)} \biggr]=0 \end{aligned}$$

because by assumption $\delta _{n}=0$. Since $\delta _{n+1}=0$, $\delta _{n+2}=0$, and so on. Therefore, $\delta _{n'}=0$ for all $n'\geq n+1$, which completes the proof. □

## Examples

We consider first the Hahn difference equation with constant coefficients

52 $$ D_{q;w}^{2}y(x) = \lambda _{0} \, D_{q;w}y(x) + s_{0}\, y(x), $$

where *a* and *b* are real constants. Although it is clear, for all $n\ge 1$, that $D_{q;w} \lambda _{n-1}=D_{q;w}s_{n-1}=0$, the terminating condition $\delta _{n}$ does not equal 0 for any fixed *n*. The ${}_{q;w}\mbox{AIM}$ sequences $\lambda _{n} = \lambda _{n-1}\, \lambda _{0} + s_{n-1}$ and $s_{n}= \lambda _{n-1}\,s_{0}$, however, yield

$$ \frac{s_{n}}{\lambda _{n}}= \frac{\lambda _{n-1}s_{0}}{ \lambda _{n-1}\lambda _{0} + s_{n-1}}. $$

Thus, as $n\to \infty $, the terminating condition reads

$$ \frac{s_{\infty }}{\lambda _{\infty }}= \frac{s_{0}}{\lambda _{0} + \frac{s_{\infty }}{\lambda _{\infty }}}. $$

This is a quadratic equation with solution given by

$$ \frac{s_{\infty }}{\lambda _{\infty }} = \frac{-\lambda _{0} \pm \sqrt{\lambda _{0}^{2}+4s_{0}}}{2}. $$

The two linearly independent solutions are then

53 $$\begin{aligned} y_{\pm }(x) &= {\prod_{i=0}^{\infty } \biggl[1-q^{i} \bigl((q-1)x+w \bigr) \frac{-\lambda _{0} \pm \sqrt{\lambda _{0}^{2}+4s_{0}}}{2} \biggr]^{-1}} \\ & = \frac{1}{ (\frac{-2\lambda _{0}\pm \sqrt{\lambda _{0}^{2}+4 s_{0}}}{2} (w+(q-1) x);q )_{\infty }}. \end{aligned}$$

As a second example, we consider the $q;w$-hypergeometric equation [28]

54 $$ D_{q;w}^{2}\, y(x)= \underbrace{- \frac{2 \,\varepsilon \, x+\gamma }{e\, x^{2}+2\, f\, x+g}}_{ \lambda _{0}(x)}\, D_{q;w}\, y(x) \underbrace{- \frac{\tau }{e\, x^{2}+2\, f\, x+g}}_{s_{0}(x)}\,y(x), $$

with the constant coefficients *ε*, *γ*, *e*, *f*, *g*, and *τ*.

Considering the terminating condition for $n=1$,

$$ \delta _{1}=\lambda _{1}s_{0}-s_{1} \lambda _{0}=\tau \, (2 \varepsilon +\tau )\equiv 0 \quad \text{if } \tau =0,-2\varepsilon . $$

Thus, for $\tau =0$, the polynomial solution $y(x)=P_{n}(x)$ is $P_{0}(x)=1$.

For $\tau =-2\varepsilon $, the exact polynomial solution, up to a multiplicative constant, is

$$ P_{1}(x) =\lim_{m\to \infty } \frac{1}{\prod_{k=0}^{m} [1+q^{k}({(1-q)\,x-w})\,\frac{s_{0}(q^{k}x+w\sum_{i=0}^{k-1}q^{i})}{\lambda _{0}(q^{k}x+w\sum_{i=0}^{k-1}q^{i})} ]}=x+ \frac{\gamma }{2\, \varepsilon }. $$

The second iteration $n=2$ yields

$$ \delta _{2}=\lambda _{2}s_{1}-s_{2} \lambda _{1}=\tau \, (2\, \varepsilon +\tau ) \bigl((1+q) (e+2\, \varepsilon )+\tau \bigr), $$

where

55 $$\begin{aligned} \textstyle\begin{cases} \lambda _{1}(x)= \frac{2\varepsilon \gamma w+\gamma (2 f+\gamma +e\, w)-g (2\varepsilon +\tau )+((e+2\varepsilon ) (\gamma +\gamma q+2\varepsilon w)-2f\tau ) x+(2 \varepsilon (e+2\varepsilon ) q-e\tau ) x^{2}}{(g+x\, (2 f+e\, x)) (g+(w+q\, x) (2 f+e\, (w+q x)))}, \\ s_{1}(x)= \frac{\tau (2 f+\gamma +(e+2\varepsilon ) w)+((1+ q)e+2\varepsilon q) \tau \, x}{(g+x\, (2 f+e\, x)) (g+(w+q\, x) (2 f+e\, (w+q x)))}, \end{cases}\displaystyle \end{aligned}$$

then $\delta _{2}=0$ if

$$ \tau =0, \qquad -2\varepsilon ,\qquad - (1+q) (e+2\varepsilon ). $$

For $\tau =- (1+q)(e+2\varepsilon )$, the second-order polynomial solution is then

$$\begin{aligned} P_{2}(x)&=\lim_{m\to \infty } \frac{1}{\prod_{k=0}^{m} [1+q^{k}({(1-q)\,x-w})\,\frac{s_{1}(q^{k}x+w\sum_{i=0}^{k-1}q^{i})}{\lambda _{1}(q^{k}x+w\sum_{i=0}^{k-1}q^{i})} ]} \\ &=x^{2}+ \frac{(2\, f+\gamma ) (1+q)+2\, \varepsilon \, w}{(1+q)\, e+2\varepsilon \, q} \,x \\ &\quad {}+ \frac{2\,\varepsilon \, g\, q+\gamma \, (2 f+\gamma +2\, \varepsilon \, w)+e\, ((1+q)\,g+\gamma \,w)}{(e+2\,\varepsilon ) ((1+q)\,e+2\,\varepsilon \, q)}. \end{aligned}$$

Next, the third iteration $n=3$ yields

56 $$ \delta _{3}=\tau (2\varepsilon +\tau ) \bigl((q+1) (e+2\varepsilon )+\tau \bigr) \bigl( \bigl(q^{2}+q+1 \bigr) \bigl((1+ q)\,e+2 \varepsilon \bigr)+\tau \bigr). $$

Thus, for

$$ \tau = - \bigl(1 + q + q^{2} \bigr) \bigl((1+q)e + 2 \varepsilon \bigr), $$

the third-order polynomial reads as follows:

$$\begin{aligned} P_{3}(x)&=x^{3}+ \frac{\tau _{2}}{q (2\,\varepsilon \, q+e\, (1+q)^{2} )} \,x^{2}+ \frac{\tau _{1}}{ (q (e (q+1)^{2}+2 \varepsilon q ) (e \sum_{i=0}^{2}q^{i} +2\varepsilon q ) )} \,x \\ &\quad {}+ \frac{\tau _{0}}{ (q ((1+q)\,e+2\varepsilon ) (2\varepsilon \, q+e (1+q)^{2} ) (2\varepsilon q+e (1+q+q^{2} ) ) )}, \end{aligned}$$

where

$$\begin{aligned} &\tau _{2}= \bigl(\gamma +2 f (1+q) \bigr)\sum _{i=0}^{2}q^{i} + \bigl(2(1+2q) \varepsilon +e (1+q) (2+q) \bigr) w, \\ &\begin{aligned} \tau _{1}&=\sum_{i=0}^{2}q^{i} \bigl(\gamma ^{2}+4\, f^{2} (1+q)+2 f \, \gamma \, (2+q)+g q \bigl(2\,\varepsilon \, q+e\, (1+q)^{2} \bigr) \bigr) \\ &\quad {}+2 \bigl((1+q)e+2\varepsilon \bigr) \sum_{i=0}^{2}q^{i} \bigl(\gamma +f (2+q) \bigr) w+2\varepsilon \bigl(2\varepsilon (1+q)+e \bigl(2+q (2+q) \bigr) \bigr) w^{2}, \end{aligned} \\ &\begin{aligned} \tau _{0}&=4\, f^{2}\, \gamma \, (1+q)+\gamma \, \bigl( \gamma ^{2}+2 \, \varepsilon \, g\, q\, (1+2 q) \bigr)+2\, \varepsilon \bigl(2\, \varepsilon \, g\, q\, (1+q)^{2}+\gamma ^{2} \, (2+q) \bigr) w \\ &\quad {}+4\,\varepsilon ^{2}\,\gamma \, (1+q)\, w^{2}+e^{2} \, (1+q)\, w\, \bigl(g\, (1+q)\, (2+q)\, \bigl(1+q+q^{2} \bigr)+ \gamma \bigl(2+q (2+q) \bigr) w \bigr) \\ &\quad {}+2 f \bigl(2\,\varepsilon \, g\, q\, (1+q)^{2}+\gamma ^{2} (2+q)+2 \varepsilon \,\gamma \bigl(2+q (2+q) \bigr) w \bigr) \\ &\quad {}+e\, \bigl(g (1+q) \bigl(\gamma +2 (1+q) \bigl(\gamma \, q+f \bigl(1+q+q^{2} \bigr) \bigr) \bigr) \\ &\quad {}+ \bigl(2\varepsilon \, g\, (1+q)^{3} (1+2 q)+\gamma \bigl(2 f (1+q) \bigl(3+q (2+q) \bigr) \\ &\quad {}+ \gamma \bigl(3+q (3+q) \bigr) \bigr) \bigr) w+2 \varepsilon \gamma \bigl(3+2 q (2+q) \bigr) w^{2} \bigr). \end{aligned} \end{aligned}$$

In general, it is not difficult to show that

57 $$ \delta _{n}=\prod_{k=0}^{n} \Biggl( \Biggl(\sum_{j=0}^{k-1} q^{j} \Biggr) \Biggl(2\, \varepsilon +e \sum _{j=0}^{k-2} q^{j} \Biggr)+ \tau \Biggr)\equiv 0, $$

and the necessary condition for the existence of the *n*-degree polynomial solution (see also [28, formula 10.1.1]) of the $(q;w)$-hypergeometric equation (54), for $n=1,2,\ldots $ , is that

58 $$ \tau _{n}=- \Biggl(\sum _{j=0}^{n-1} q^{j} \Biggr) \Biggl(2\, \varepsilon +e \sum_{j=0}^{n-2} q^{j} \Biggr). $$

With the ${}_{q;w}\mbox{AIM}$ technique under disposal, we hope it will be useful to study the analytic and approximate solutions of the *q*-difference equation. It will also be a practical tool in the construction of *q*-orthogonal polynomials and deepen our understanding of their mathematical properties.

## Data Availability

Not applicable.
